# Clinical application of chromosomal microarray analysis for the diagnosis of Williams–Beuren syndrome in Chinese Han patients

**DOI:** 10.1002/mgg3.517

**Published:** 2018-12-18

**Authors:** Yu Xia, Shufang Huang, Yueheng Wu, Yongchao Yang, Shaoxian Chen, Ping Li, Jian Zhuang

**Affiliations:** ^1^ Department of Cardiovascular Surgery of Guangdong Provincial Cardiovascular Institute, Guangdong General Hospital Guangdong Academy of Medical Sciences Guangdong China; ^2^ Prenatal Diagnosis Center, Guangdong General Hospital Guangdong Academy of Medical Sciences Guangdong China; ^3^ Guangdong Provincial Key Laboratory of South China Structural Heart Disease, Guangdong General Hospital Guangdong Academy of Medical Sciences Guangdong China; ^4^ Department of Obstetrics and Gynecology, Guangdong General Hospital Guangdong Academy of Medical Sciences Guangdong China; ^5^ Research Department of Medical Sciences, Guangdong General Hospital Guangdong Academy of Medical Sciences Guangdong China

**Keywords:** chromosomal microarray analysis, congenital heart disease, copy number variation, facial dysmorphism, Williams‐Beuren syndrome

## Abstract

**Background:**

Williams–Beuren syndrome (WBS; OMIM #194,050) is a rare multisystem disorder of a variable phenotypic spectrum caused by a heterozygous microdeletion in the WBS chromosome region (WBSCR) in 7q11.23.

**Methods:**

We screened 38 Chinese Han patients with suspected WBS using chromosomal microarray analysis (CMA).

**Results:**

Pathogenic CNVs were identified in 34 of the patients, including 29 cases with a typical 7q11.23 microdeletion, three cases with atypical copy number variations (CNVs) within the WBS chromosome region and two cases with CNVs associated with other known syndromes. All 29 WBS patients with a typical microdeletion exhibited distinctive facial dysmorphisms and developmental delay. We observed that the incidence of pulmonary abnormalities was slightly higher than that of aortic abnormalities. We also found long philtrum and prominent lips with a thick lip that may warrant suspicion of WBS in the Chinese Han patients.

**Conclusion:**

CMA facilitates diagnosis in individuals with classic/nonclassic features of WBS and demonstrated that when Chinese Han patients present with a less classical phenotype, such as pulmonary abnormalities, this may raise suspicion for a WBS diagnosis and suggest a referral for a genetics evaluation for a differential diagnosis.

## Abstract 2

Our study demonstrates that although the clinical features of WBS display a highly variable phenotypical spectrum, CMA facilitates diagnosis in individuals with classical and nonclassical features of WBS. In Chinese patients, a less classical phenotype in other races and ethnicities, such as PAS, long philtrum, and global developmental delay, should raise suspicion for WBS and suggest referral for a genetics evaluation and a differential diagnosis.

## 1 INTRODUCTION

Williams–Beuren syndrome (WBS; OMIM #194050) is a rare multisystem disorder with a variable phenotypic spectrum that includes a distinctive facial appearance, cardiovascular abnormalities, developmental delays, and aberrant neurocognitive profile (Pober, 2010). The prevalence of WBS is estimated to be one in 7,500 to 20,000 live births (Dutly & Schinzel, 1996; Strømme, Bjømstad, & Ramstad, 2002).

Williams–Beuren syndrome is caused by a heterozygous microdeletion in the WBS chromosome region (WBSCR) on chromosome 7 at position 7q11.23. The common deletion/duplication ranges in size from 1.55 to 1.84 Mb and spans approximately 26–28 genes. This region is flanked by low copy repeats (LCRs) that can mediate nonallelic homologous recombination resulting from a misalignment of LCRs during meiosis (Savina et al., 2011). Moreover, clear Mendelian inheritance for WBS has been observed in a small proportion of patients with a family history of the disease (Parlak et al., 2014).

Clinical diagnosis of WBS is made based on dysmorphic features, aortic anomalies, intellectual findings; however, the broad spectrum of anomalies exhibits variable expressivity. In some specific conditions, such as Noonan syndrome and Turner syndrome, may present with similar symptoms that may be confused with WBS. An atypical CNV may be the leading cause of the substantial phenotypic variability among WBS patients, thereby making this disease difficult to classify and diagnose in some cases. Thus, frequently a genetic test is necessary to confirm a diagnosis.

In the past, the American College of Medical Genetics and Genomics (ACMG) recommends the use of fluorescence in situ hybridization (FISH) as the first‐tier diagnostic test for WBS (Manning & Professional, 2010). However, this approach is not suitable for the study of CNV due to low‐resolution and low throughput (Hussein et al., 2016). More recently, Multiplex ligation‐dependent probe amplification (MLPA) and chromosomal microarray analysis (CMA) have been successfully applied to detect the CNV in the 7q11.23 regions (Honjo et al., 2015; Li et al., 2016; Sharma et al., 2015). Exploring the underlying genetic etiology of CNV in WBS patients may provide more information about diagnosis, such as CNV length and affected genes. These genetic data also have proven to be an invaluable source for understanding how haploinsufficient genes contribute to disease pathogenesis.

In this study, we reviewed the clinical and molecular findings in 38 Chinese Han patients with clinically suspected WBS to explore the molecular etiology and to assess the clinical significance of each factor. We analyzed the clinical features and genetic data and elucidated the genotype–phenotype correlations in 29 patients with classically presenting WBS. We further assessed the clinical significance of the genetic results in patients presenting with similar symptoms to WBS to provide more information for clinical screening and genetic counseling for the disease.

## 2 MATERIALS AND METHODS

### 2.1 Study subjects

We analyzed a cohort of 38 Chinese Han patients (15 females and 23 males) with suspected WBS from the Prenatal Diagnosis Center and the Department of Cardiac Surgery of the Guangdong General Hospital between July 2015 and March 2018. All patients underwent a complete examination according to the Genetics Committee of the American Academy of Pediatrics (2001), and those receiving a score ≥3 were enrolled in the study (Leme et al., 2013). Clinical data, including medical records, electrocardiograms, echocardiography, and cardiac catheterization reports were systematically reviewed. The family history was obtained by interviewing the parents of the index cases. The study protocol was approved by the Institutional Research Ethics Committee of the Guangdong General Hospital. Informed written consent was obtained from the patients’ parents. Approximately 2.0 ml of peripheral venous blood was collected, and DNA was extracted with the Gentra Puregene blood kit (QIAGEN, Santa Clara, CA, USA) according to the manufacturer's instructions.

### 2.2 Fluorescent in situ hybridization

We have performed FISH in five patients. FISH was carried out on metaphase spreads using the dual color locus‐specific identifier WBS region probe (Vysis probe; Abbot, USA) that hybridizes to the *ELN*, *LIMK1,* and *EIF4H* loci at 7q11.23 (orange) and the control loci D7S486 and D7S522 at 7q31 (green). Approximately 10–15 cells in metaphase were analyzed for each patient.

### 2.3 Chromosomal microarray testing and CNV evaluation and validation

DNA (250 ng) was amplified, labeled, and hybridized to the CytoScan HD array platform (Affymetrix, USA) according to the manufacturer's protocol. Data were visualized and analyzed with the Chromosome Analysis Suite (ChAS) software package (Affymetrix, USA) with a minimal cutoff of 20 consecutive markers in a 25‐kb length for CNV calling. All segments were monitored for the degree of overlap with previously identified common CNVs and annotated by the Database of Genomic Variants (DGV). All reported CNVs were based on NCBI human genome build 37 (hg 19).

Detected CNVs meeting the following criteria were selected for further analysis: (1) deletions ≥50 kb/25 markers; duplications ≥100 kb/50 markers; (2) not found in the control populations cataloged in the DGV; and (3) <50% overlap with known segmental duplications (SD).

A total of 174 population‐based controls without cardiac lesions were selected from the local database. Additional controls included DECIPHER (https://decipher.sanger.ac.uk/), the DGV (https://dgv.tcag.ca/dgv/app/home), the 1000 Genomes Project (https://www.1000genomes.org/), the Deciphering Developmental Disorders (DDD) Project (https://www.ddduk.org/), and previously published studies that used high‐density microarray platforms comparable to the ones used in this study.

Following the ACMG's standards and guidelines for the interpretation of CNVs, the remaining CNVs were classified into three categories: pathogenic (P), variants of uncertain significance (VUS), and benign (B). VUS was further divided into three parts: likely pathogenic (LP), likely benign (LB), and no subclassification (Kearney, Thorland, Brown, Quintero‐Rivera, & South, 2011). Only genes that function in a dominant manner that are within the pathogenic CNVs and likely pathogenic CNVs were investigated in this study.

All annotated CNVs were experimentally validated by real‐time quantitative PCR (qPCR). During the initial period of the study, five patient samples tested by FISH were also detected by CMA.

### 2.4 Statistical analysis

Statistical analysis was performed with SPSS 23.0 (SAS Institute, Cary, NC, USA). The data collected were expressed with mean ± *SD*. A *p* value of <0.05 was considered statistically significant.

## 3 RESULTS

### 3.1 Patients’ demographics and clinical characteristics

Between July 2015 and March 2018, 38 patients with suspected WBS were eligible for inclusion in the study and underwent CMA. The average patient age at diagnosis was 24.2 months (14.2–34.1, 95%). In total, 39.5% (15/38) were female and 60.5% (23/38) were male. All patients without a family history of WBS.

### 3.2 FISH findings in five suspected WBS cases

FISH analysis indicated the *ELN* deletion in all the five suspected WBS cases (Supporting Information Figure S1).

### 3.3 CMA findings in 38 suspected WBS cases

An interpretable CMA profile was obtained for all of the 38 genomic DNA samples. Seven CNVs identified in nine patients were considered to be likely benign, listed in the DGV or of no known gene included. We also found 6 (15.7%) patients with VUS CNVs.

Pathogenic CNVs were detected in 34 of 38 samples (89.5%) that overlapped with well‐characterized WBS CNVs, with a DECIPHER entry, or comprised OMIM genes. These included 29 cases with a typical 7q11.23 microdeletion ranging from 1.4 to 1.9 Mb (see Table 1). We also identified three atypical CNVs associated with WBS, including one case with a compound atypical microdeletion and microduplication within the WBSCR, one case with a smaller atypical microdeletion involving the *ELN*, and one case with a typical microduplication in 7q11.23 (see Table 2). In addition, two cases presented with CNVs associated with other known syndromes, including 22q11.2 microdeletion syndrome (22q11DS, DiGeorge syndrome) and 10p15.3 microdeletion. (see Table 3). Of these 34 patients, 10.5% (4/38) had more than one CNV. Notably, there were 10.5% (4/38) cases for which no abnormalities were detected by CMA.

**Table 1:** Typical 7q11.23 microdeletion in the WBSCR identified in classical WBS patients by CMA

No.	Sex	Age (month)	Cytoband	Distinctive facies	Cardiovascular disease	Intellectual disability	Growth abnormalities	Motor abnormalities	Endocrine abnormalities
1	M	8	Del 7q11.23 (72,668,413–74,242,132)	+	+	N/A	+	−	−
2	F	36	Del 7q11.23 (72,702,149–74,142,256)	+	+	+	+	+	+
3	M	30	Del 7q11.23 (72,364,514–73,777,326)	+	+	+	+	+	+
4	M	24	Del 7q11.23 (72,700,996–74,142,256)	+	+	+	+	+	−
5	M	18	Del 7q11.23 (72,718,277–74,143,060)	+	+	+	+	−	−
7	F	30	Del 7q11.23(72,701,098–74,186,150)	+	−	+	−	−	−
8	M	18	Del 7q11.23(72,470,639–74,287,433)	+	+	+	−	−	−
10	M	15	Del 7q11.23 (72,720,001–74,142,190)	+	+	+	+	+	−
11	F	6	Del 7q11.23 (72,589,515–74,289,484)	+	+	N/A	−	−	−
12	M	84	Del 7q11.23 (72,701,018–74,142,190)	+	−	+	+	−	−
16	F	31	Del 7q11.23 (72,590,362–74,149,104)	+	+	+	+	+	−
17	M	5	Del 7q11.23 (72,758,096–74,149,104)	+	+	N/A	+	+	−
18	M	8	Del 7q11.23 (72,701,098–74,136,633)	+	+	N/A	+	−	−
			Dup 14q12 (24,626,462–27,898,553)						
20	M	31	Del 7q11.23 (72,624,166–74,209,678)	+	+	+	+	−	−
21	M	18	Del 7q11.23 (72,329,724–74,628,840)	+	+	+	+	+	−
23	M	1	Del 7q11.23 (72,701,018–74,142,190)	+	+	N/A	+	N/A	−
24	M	21	Del 7q11.23 (72,700,996–74,142,256)	+	+	+	−	+	−
25	F	16	Del 7q11.23 (72,621,722–74,142,190)	+	+	+	+	+	−
26	M	4	Del 7q11.23 (72,717,535–74,115,002)	+	+	N/A	−	N/A	−
27	M	8	Del 7q11.23 (72,692,112–74,154,209)	+	+	N/A	+		−
28	F	36	Del 7q11.23 (72,645,834–74,172,862)	+	+	+	+	−	−
			Del 6p25.3 (1,332,312–1,858,186)						
30	F	32	Del 7q11.23 (72,590,362–74,136,747)	+	−	+	+	+	+
31	F	37	Del 7q11.23 (72,611,954–74,298,268)	+	+	+	+	+	+
33	M	8	Del 7q11.23 (72,749,941–74,136,633)	+	+	N/A	+	+	+
34	M	6	Del 7q11.23 (72,642,158–72,292,158)	+	+	N/A	+	+	+
35	M	8	Del 7q11.23 (72,718,277–74,142,190)	+	+	N/A	+	+	−
36	F	120	Del 7q11.23 (72,632,294–74,142,190)	+		+	+	−	+
37	M	24	Del 7q11.23 (72,745,738–74,129,824)	+	+	+	−	+	−
38	M	60	Del 7q11.23 (72,589,600–74,287,433)	+	+	+	−	+	−

Note

N/A, not available.

**Table 2:** Atypical 7q11.23 CNV in the WBSCR identified in nonclassical WBS patients by CMA

ID	Sex	Age (month)	Cytoband	Length	RefSeq genes	Phenotypes
13	M	60	Dup 7q11.23 (72,470,639–74,438,633)	1.97 Mbp	***NSUN5 TRIM50 FKBP6 FZD9 BAZ1B BCL7B TBL2 MLXIPL VPS37D DNAJC30BUD23 STX1A ABHD11 CLDN3 CLDN4 METTL27 TMEM270 ELN LIMK1 EIF4H LAT2 RFC2 CLIP2*** *** GTF2IRD1 LOC107986810 GTF2I NCF1 GTF2IRD2 GATSL1***	pulmonary artery stenosis, coarctation of the aorta, intellectual disability
15	M	31	Del 7q11.23 (73,467,492–73,474,831)	7,340 bp	*ELN* *(exons* *18* *–* *25)*	pulmonary artery stenosis, left pulmonary artery stenosis, right pulmonary artery stenosis, developmental delay
29	F	8	Dup 7q11.23 (72,221,649–73,349,597)	1.13 Mbp	***TYW1B POM121 TRIM74 NSUN5 TRIM50 FKBP6 FZD9 BAZ1B BCL7B TBL2 MLXIPL VPS37D DNAJC30BUD23 STX1A ABHD11 CLDN3 CLDN4***	distinctive facial appearance, pulmonary artery stenosis, developmental delay
			Del 7q11.23 (73,433,055–74,339,045)	0.91 Mbp	*ELN LIMK1 EIF4H LAT2 RFC2 * *CLIP2* * GTF2IRD1 LOC107986810 GTF2I NCF1 GTF2IRD2 GATSL1*	

Note

Genes in bold are reported to be duplication; genes in light are reported to be deletion.

**Table 3:** Known pathogenic CNVs identified in patients suspected to WBS by CMA

ID	Sex	Age (month)	Cytoband	Length	RefSeq genes	Phenotypes
14	M	2	Del 10p15.3p15.1:(120,001–6,540,000)	6.42 Mbp	ZMYND11 DIP2C GTPBP4 IDI1 WDR37 ADARB2 PFKP PITRM1 KLF6 AKR1E2 1KR1C3 TUBAL3 NET1 TUBAL3 ASB13 FAM208B ANKRD16 IL15RA GDI2 FBX018 PFKFB3 PRKCQ	long philtrum, short nose with anteverted nares, periorbital puffiness, ocular hypertelorism, atrial septal defect, congenital talipes equinovarus, developmental delay, intellectual disability
			Dup 12q24.31q24.33(125,280,001–133,820,000)	8.54 Mbp	**Scarb1 ubc dhx37 bri3bp aacs tmem132b slc15a4 glt1d1 tmem132d fzd10 piwil1 stx2 gpr133 sfswap mmp17 ulk1 pus1 ep400 ddx51 noc4l galnt9 fbrsl1 lrcol1 p2rx2** **pole pxmp2 pgam5 ankle2 golga3 chfr znf605 znf26 znf84 znf140 znf268 anhx**	
19	M	2	Del 22q11 (18,893,887–21,386,101)	2.49 Mbp	*DGCR6 PRODH DGCR2 DGCR14 TSSK2 GSC2 SLC25A1 CLTCL1 HIRA MRPL40 C22orf39 UFD1 CDC45 CLDN5 Sept5‐GP1BB TBX1 GNB1L RTL10 TXNRD2 COMT ARVCF TANGO2 DGCR8 TRMT2A RANBP1 ZDHHC8 CCDC188 RTN4R DGCR6L USP41 ZNF74 SCARF2 KLHL22 MED15 PI4KA SERPIND1 SNAP29 CRKL AIFM3 LZTR1 THAP7 P2RX6 SLC7A4 LRRC74B GGT2 RIMBP3B HIC2*	patent duct artery, left pulmonary artery stenosis, bilateral indirect inguinal hernia, right crumpled ear, hypocalcemia

Note

Genes in bold are reported to be duplication; genes in light are reported to be deletion.

### 3.4 Clinical features in classical and nonclassical WBS patients

Based on the clinical presentation and CMA results, 29 patients with a typical microdeletion in the WBSCR were diagnosed with classical WBS. Clinical features were analyzed in these 29 classical WBS patients (the patient with an atypical 7q11.23 CNV was excluded). The average classical WBS patients’ age at diagnosis was 25.7 months (15.9–35.5, 95%). In total, 27.6% (8/29) were female and 72.4% (21/29) were male. The findings are summarized in Table 1.

All classical WBS patients exhibited a distinct facial appearance (Supporting Information Figure S2). The craniofacial features of classical WBS patients included a long philtrum (27/29), prominent lips with a thick lip (26/29), short nose with anteverted nares (25/29), periorbital puffiness (24/29), ocular hypertelorism (24/29), and abnormal teeth (18/21) (Table 4).

**Table 4:** Facial Features in 29 classical WBS patients

Facial features	Frequency	Percentage (%)
Long philtrum	27/29	93.1
Prominent lips with a thick lip	26/29	89.7
Short nose with anteverted nares	25/29	86.2
Periorbital puffiness	24/29	82.8
Ocular hypertelorism	24/29	82.8
Abnormal teeth	18/21	62.1

The frequency of cardiac abnormalities in classical WBS patients was 89.7% (26/29) (Table 5). The cardiac abnormalities were pulmonary abnormalities (17/29), aortic defects (15/29), and intracardiac lesions (12/29). The most frequent pulmonary findings in our WBS patients were pulmonary artery stenosis (PAS; 9/29), pulmonary valve stenosis (PVS; 4/29), left pulmonary artery stenosis (LPAS; 5/29), and right pulmonary artery stenosis (RPAS; 5/29). Fifteen patients exhibited aortic diseases, including supravalvular aortic stenosis (SVAS; 7/29), coarctation of the aorta (CoA; 6/29), and aortic valve stenosis (AVS; 4/29). Overall, 41.4% of cases (12/29) were affected by “left‐right shunt CHDs,” including atrial septal defects (ASD; 7/29), ventricular septal defects (VSD; 4/22), patent foramen ovale (PFO; 2/29), patent ductus arteriosus (PDA; 2/29), and 55.2% of cases (17/29) were not annotated. Isolated SVAS with no other cardiac lesion was observed in three patients. We also found one patient had arrhythmia.

**Table 5:** Type of cardiac malformation in 29 classical WBS patients

Cardiac Malformation	Frequency	Percentage (%)
Aortic defects		
SVAS	7/29	24.1
CoA	6/29	20.7
AVS	4/29	13.8
Pulmonary abnormalities		
PAS	9/29	31.0
PVS	4/29	13.8
LPAS	5/29	17.2
RPAS	5/29	17.2
Left‐right shunt CHDs		
ASD	7/29	24.1
VSD	4/22	13.8
PFO	2/29	6.9
PDA	2/29	6.9

Note

ASD, atrial septal defects; AVS, aortic valve stenosis; CoA, coarctation of the aorta; LPAS, left pulmonary artery stenosis; PAS, pulmonary artery stenosis; PDA, patent ductus arteriosus; PFO, patent foramen ovale; PVS, pulmonary valve stenosis; RPAS, right pulmonary artery stenosis; SVAS, supravalvular aortic stenosis; VSD, ventricular septal defects.

Among classical WBS patients, nineteen (19/29) presented with intellectual disability (10 classical WBS patients under 1 year old were unavailable for testing). Developmental delay was observed in 62.1% of patients (18/29). Motor developmental delay was also present in 55.2% of patients (16/27). Other relatively less common findings in our study included hypothyrea (2/29), hyperthyrea (3/29), inguinal hernia (2/29), hypercalcemia (1/29), and hypertension (1/29), based on the available records.

Moreover, we identified two patients with an atypical microdeletion in the WBSCR. These patients were diagnosed with nonclassical WBS. One patient (No. 29) with a compound deletion and duplication in 7q11.23, this patient presented with a distinctive facial appearance, PAS, and developmental delay. Another patient (No. 15) with a smaller microdeletion involving deletion of exons 18–25 in ENL gene, presented with isolated PAS, LPAS, RPAS, and developmental delay, but no other WBS‐related syndromes (Table 2).

Furthermore, we identified one patient (No. 13) with a 1.8 Mb microduplication in the WBSCR. This patient was diagnosed with 7q11.23 microduplication syndrome. He was affected by PS and CoA at birth and subsequently presented with profound intellectual disability and atypical behavior (Table 2).

### 3.5 Clinical features in two patients with CNVs associated with known syndromes

We also identified one patient with a 22q11 microdeletion, who presented with PAS, distinctive facial features, bilateral Indirect Inguinal Hernia, right crumpled ear, and hypocalcemia. Another patient with a 10p15.3 microdeletion presented with distinctive facial features, ASD, congenital talipes equinovarus (CTEV), developmental delay, and intellectual disability (Table 3).

## 4 DISCUSSION

In this study, we detected clinically significant results in 89.5% (34/38) of cases suspected to have WBS. We identified 29 patients with classical WBS characterized by a typical deletion, two patients with nonclassical WBS characterized by atypical CNVs in 7q11.23 and one patient with a 7q11.23 duplication. Our results are in agreement with previous reports showing that a CMA approach can robustly identify pathogenic CNVs in WBS. These findings indicate that CMA is an important supplement to clinical examination for accurate diagnosis of WBS (Hussein et al., 2016). Notably, one patient with a small CNV involving the *ELN*, presented with isolated PAS, LPAS and RPAS (see Table 2). Previous studies have shown that *ELN* contributes mainly to elastic fiber formation and confers elasticity to organs and tissues; deletions and mutations in this gene are associated with SVAS. These CMA findings provide possible clues to further explore the mechanisms underlying the association between *ELN* deletion and pulmonary artery defects. Further elucidating the genetic basis of WBS‐associated genes will expand our understanding of the disease etiology. Moreover, we identified two pathogenic CNVs related to known syndromes, such as 22q11 DS and 10p15.3 microdeletion syndrome. The clinical features of these genetic syndromes can be easily confused with WBS, suggesting that if the CMA is combined with clinical examination, a more accurate diagnosis can be achieved. Nevertheless, 10.5% (4/38) of the cases remained elusive, suggesting that other factors may be implicated in the etiology of those patients.

The facial dysmorphic features were present in all patients with typical deletion (*n* = 29) and displayed a distinct pattern. The most prominent facial features were a long philtrum (27/29), prominent lips with a thick lip (26/29), and short nose with anteverted nares (25/29). These findings are not entirely consistent with previous reports in other populations (Ferrero et al., 2007; Kruszka et al., 2018). Patil, Madhusudhan, Shah, and Suresh (2012) reported that a wide mouth, short nose, and periorbital fullness were the most prominent facial features (27/27), and long philtrums were found in 63.4% of Indian patients (23/27). Viana, Stofanko, Gonçalves‐Dornelas, da Silva Cunha, and de Aguiar (2013) demonstrated that Brazilian patients had prominent lips (14/15), slanted palpebral fissures (9/15), and a long philtrum (8/15). Using both clinical exam and facial analysis technology, Kruszka et al. (2018) found wide mouth, short nose, and texture of eyelids/epicanthic folds were the common characteristic features of WBS in the global population, but the width of the mouth was not depicted as a top feature of WBS in the African group. We consider that the discrepancies in facial morphology among different populations are likely due in part to differences in genetic background of different populations and ethnicities. Thus, a long philtrum and prominent lips with a thick lip are facial anomalies that may warrant suspicion of WBS in the Chinese Han population.

Great arteries anomalies were observed in 72.4% of patients (21/29). These results are consistent with previous reports of 66%–85% incidence in WBS patients (Rubens, Rodríguez, Hach, DelCastillo, & Martínez, 2008; Yuan, 2017). However, we observed the incidence of pulmonary abnormalities (17/29) to be slightly higher than that of aortic abnormalities (15/29) in the Chinese Han population, which is consistent with what was reported by Yau, Lo, and Lam (2004) but not with what was reported in previous studies on other populations (Ferrero et al., 2007; Rubens et al., 2008; Yuan, 2017). We also found PAS was the most common cardiovascular defect (9/29), followed by SVAS (7/29) and ASD (7/29). Cases of “left‐right shunt CHDs” were frequent among our patients (12/29). Interestingly, 10.3% of our WBS patients did not exhibit a cardiac phenotype. We speculate that prenatal diagnosis, such as fetal echocardiography and fetal CMA, contribute to a reduced number of WBS patients with cardiovascular abnormalities, particularly in aortic abnormalities associated with WBS. If fetuses do not manifest heart defects, their parents will not seek further genetic testing. Therefore, the numbers of WBS patients without cardiovascular abnormalities increased. Accordingly, it is necessary to improve genetic testing, provide genetic counseling, and to raise public awareness of WBS for parents to reduce birth rate of WBS.

In our study, all patients had various degrees of physical and mental disabilities, as previously described (Hussein et al., 2016; Saad et al., 2013; Sharma et al., 2015). Other relatively less common findings in our study included hypothyrea/hyperthyrea, hypercalcemia, inguinal hernia, and strabismus. These observations were concordant with the prevalence and variations described in the literature (reported to occur in 5%–10% of patients; Sammour et al., 2014; Sindhar et al., 2016).

Today, FISH and MLPA remain the most widely used laboratory tests for WBS diagnosis (Dutra et al., 2012; Hussein et al., 2016; Manning & Professional, 2010; Sharma et al., 2015). However, compared to FISH and MLPA, CMA is not only suitable for identifying typical/atypical CNVs and refining the distal break point for classical or nonclassical WBS, but also can identify other potential pathogenic CNVs, due to its high‐resolution and high accuracy at the whole genome level. Due to their relative simplicity and time efficiency, FISH and MLPA are better suited for a primary genetic screen for WBS. In addition, it is necessary to improve genetic counseling, to develop more detection approaches, especially for assessing brain morphology and function, and to raise public awareness of WBS to increase the detection rate.

The present study has some limitations. First, the intellectual development of these WBS patients could not be accurately assessed because the average patient age was <3 years. Second, we did not have information about CNVs for all the patients’ parents. Thus, only some genetic information related to CNVs was obtained.

In summary, our study demonstrates that although the clinical features of WBS display a highly variable phenotypical spectrum, CMA facilitates diagnosis in individuals with classical and nonclassical features of WBS. In Chinese Han patients, a less classical phenotype in other races and ethnicities, such as PAS and long philtrum should raise suspicion for WBS and suggest referral for a genetics evaluation and a differential diagnosis.

## CONFLICT OF INTEREST

All authors declare no conflict of interest.
