# Supplementary material for: Clinical application of chromosomal microarray analysis for the diagnosis of Williams–Beuren syndrome in Chinese Han patients
Source: Mol Genet Genomic Med. 2018 Dec 18;7(2):e00517. doi: 10.1002/mgg3.517 (PMC6393686; doi:10.1002/mgg3.517)
Supplement: Supplementary file 1 [file MGG3-7-na-s001.pdf]

Supplement Fig. 1. FISH results in WBS patients and healthy individual

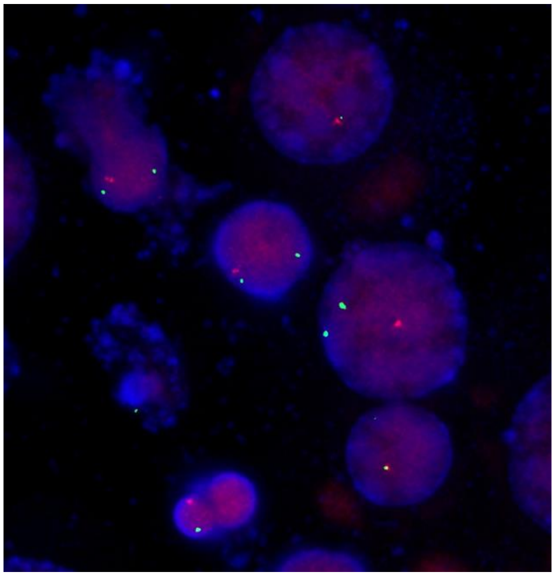

WBS patients

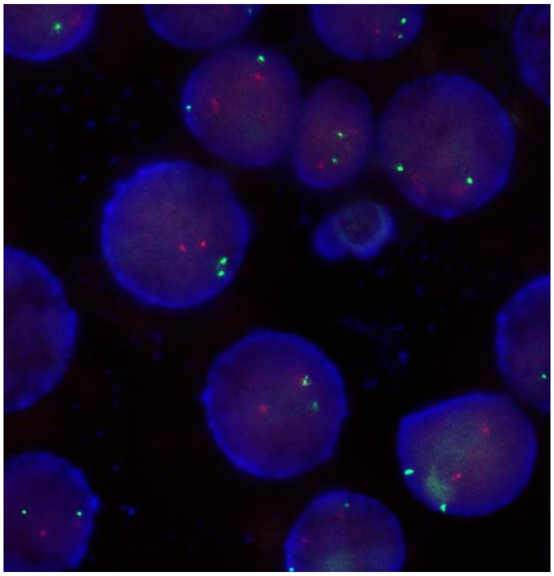

Healthy individual
