# Supplementary material for: Clinical application of chromosomal microarray analysis for the diagnosis of Williams–Beuren syndrome in Chinese Han patients
Source: Mol Genet Genomic Med. 2018 Dec 18;7(2):e00517. doi: 10.1002/mgg3.517 (PMC6393686; doi:10.1002/mgg3.517)
Supplement: Supplementary file 2 [file MGG3-7-na-s002.pdf]

Supplement Fig. 2. Classical Williams-Beuren syndrome patient exhibited a distinct facial appearance.

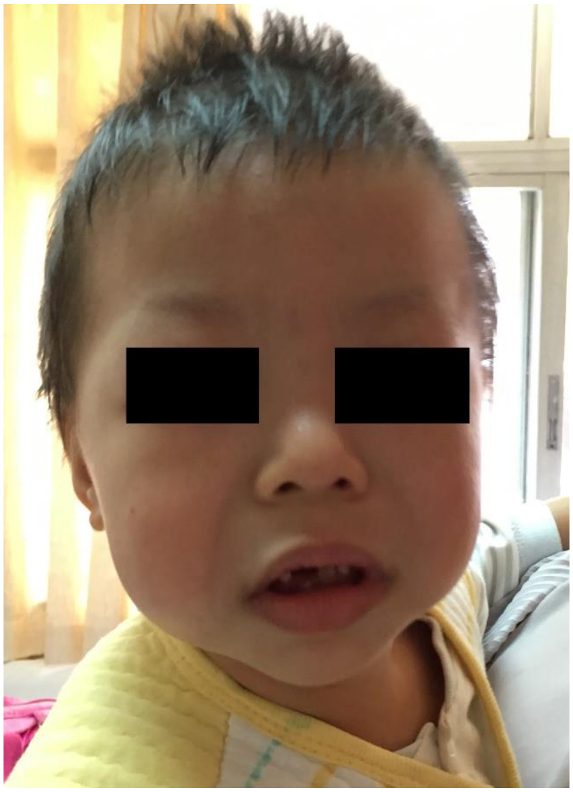

Supplement Fig. 2. Clinical photograph of one WBS patient. At 19 months of age. Note full cheeks, broad forehead with mild frontal bossing, ocular hypertelorism, short nose with anteverted nares, depressed nasal bridge, short nose, long philtrum, prominent lips with a thick lip, abnormal teeth and micrognathia.
